# Divergent responses to peptidoglycans derived from different *E. coli *serotypes influence inflammatory outcome in trout, *Oncorhynchus mykiss*, macrophages

**DOI:** 10.1186/1471-2164-12-34

**Published:** 2011-01-14

**Authors:** Sebastian Boltaña, Felipe Reyes-Lopez, Davinia Morera, Frederick Goetz, Simon A MacKenzie

**Affiliations:** 1 Institute of Biotechnology and Biomedicine, Universitat Autónoma de Barcelona, 08193 Barcelona, Spain; 2 Departamento de Biologia, Universidad de Santiago de Chile, Santiago de Chile, Chile; 3 Great Lakes WATER Institute, 600 E. Greenfield Ave., Milwaukee, WI 53204, USA

## Abstract

**Background:**

Pathogen-associated molecular patterns (PAMPs) are structural components of pathogens such as lipopolysaccharide (LPS) and peptidoglycan (PGN) from bacterial cell walls. PAMP-recognition by the host results in an induction of defence-related genes and often the generation of an inflammatory response. We evaluated both the transcriptomic and inflammatory response in trout (*O. mykiss*) macrophages in primary cell culture stimulated with DAP-PGN (DAP; meso-diaminopimelic acid, PGN; peptidoglycan) from two strains of *Escherichia coli *(PGN-K12 and PGN-O111:B4) over time.

**Results:**

Transcript profiling was assessed using function-targeted cDNA microarray hybridisation (n = 36) and results show differential responses to both PGNs that are both time and treatment dependent. Wild type *E. coli *(K12) generated an increase in transcript number/diversity over time whereas PGN-O111:B4 stimulation resulted in a more specific and intense response. In line with this, Gene Ontology analysis (GO) highlights a specific transcriptomic remodelling for PGN-O111:B4 whereas results obtained for PGN-K12 show a high similarity to a generalised inflammatory priming response where multiple functional classes are related to ribosome biogenesis or cellular metabolism. Prostaglandin release was induced by both PGNs and macrophages were significantly more sensitive to PGN-O111:B4 as suggested from microarray data.

**Conclusion:**

Responses at the level of the transcriptome and the inflammatory outcome (prostaglandin synthesis) highlight the different sensitivity of the macrophage to slight differences (serotype) in peptidoglycan structure. Such divergent responses are likely to involve differential receptor sensitivity to ligands or indeed different receptor types. Such changes in biological response will likely reflect upon pathogenicity of certain serotypes and the development of disease.

## Background

Detection of pathogens by host organisms requires direct contact between host PRRs (pattern recognition receptors) and pathogen-associated molecular patterns (PAMPs) where PAMP-PRR interactions subsequently dictate the development of the host immune response [1,2]. PAMPs such as the lipopolysaccharides (LPS) and peptidoglycans (PGN), both bacterial cell wall components, have been directly implicated in the induction of the host immune response across the vertebrata [3-9]. Peptidoglycan and related fragments are recognised by the host and induce diverse biological effects, including inflammation, leukocytosis, or enhanced immune responses [10-13]. Like LPS, peptidoglycan, including its minimal immunomodulatory subunit, muramyl dipeptide, can bind to the CD14 receptor of target cells in mammals [14-16] although peptidoglycan does not bind to LBP or BPI [17,18]. PGN's do not activate TLR4-mediated signal transduction but do activate both the TLR2 and NOD pathways [19-24].

In Drosophila, PGN recognition is achieved by the Toll or Immune deficiency (Imd) pathways, at least in part, through peptidoglycan recognition proteins (PGRPs) [25,26]. Both pathways share common features with mammalian Toll-like receptor (TLR) and tumour necrosis factor-α (TNF-α) receptor signalling cascades that regulate NF-κB activation [27-29]. In vivo studies in the zebrafish have shown that the PGRP response is essential for successful responses to bacterial infection [30]. Recently, PGN in trout macrophages has been shown to be the major stimulatory component in crude LPS preparations characterised by an increase in cytokine mRNAs, IL-1β and IL-6, and release of inflammatory products as prostaglandin E_2 _(PGE_2_) [9]. However, studies addressing different responses to serotype-specific PGNs are scarce throughout the vertebrata including mammals.

PGN may account for approximately one-half of the cell wall mass in gram-positive bacteria whereas in gram-negative bacteria only a relatively thin PGN layer in the periplasmic space is present [31,32]. Gram-negative peptidoglycan contains meso-diaminopimelic acid (DAP) as the major peptide group that is directly cross-linked whereas most gram-positive bacteria have L-lysine as the third amino acid (Lys-type). These Lys-type peptides are cross-linked through an inter-peptide bridge that varies in length and amino acid composition in different bacteria [32-34].

As the structure and composition of the microbial motif has an important role in host sensing and minor modifications in structure can influence the immune response [35-38] we explored the response of differentiated trout macrophages in cell culture to different PGNs from *E. coli *of different strains (K12 and O111:B4). Our results show that trout macrophages differentially respond to different PGNs at the level of the transcriptome by either differentially activating RNA transcripts related to prostaglandin synthesis resulting in the liberation of prostaglandin's (PGN-O111:B4) or by generating a non-defined inflammatory response,(PGN-K12).

## Methods

### Animals and Materials

Healthy adult specimens (160 g mean weight) of rainbow trout (*O. mykiss*) were purchased from a commercial hatchery (Piscifactoria Andrés, St Privat, Girona) and held in recirculating freshwater stock tanks (300 L) in the aquarium facilities at the Universitat Autònoma de Barcelona. Fish were kept at 15°C with a 12 h light/12 h dark photoperiod cycle, and were fed with a maintenance ratio of about 0.5% body weight per day. Water quality indicators (dissolved oxygen, ammonia, nitrite, pH) were analysed periodically.

DMEM and FBS were purchased from PAA Laboratories (Spain). Poly-D-lysine was purchased from Sigma (Tres Cantos, Madrid). Primocin, and PGN preparations (PGN *E. coli *K12, O111:B4) were purchased from Invivogen (Nucliber, Spain). Cell strainers and plasticware were purchased from BD Biosciences (Madrid, Spain). Gel Green was purchased from Biotium (Labnet, Spain). Prostaglandin E_2 _and D_2 _enzyme immunoassay (EIA) kit was from Cayman (Scharlab, Spain).

### Cell culture and stimulation

The experimental protocols used for head kidney isolation have been reviewed and approved by the Ethics and Animal Welfare Committee of the Universitat Autonoma de Barcelona, Spain. After anaesthetising the fish in 3-aminobenzoic acid ethyl ester (0.1 g/L), animals were sacrificed and the head kidney was dissected out. Trout macrophages were isolated as previously described [39]. Before stimulation, differentiated macrophages were incubated in serum free medium for 3 h. For stimulation, the medium of each well was removed and fresh medium containing the indicated concentrations of PGN were added and the cultures were incubated for the indicated times.

### RNA extraction and complementary DNA (cDNA) synthesis

Total RNA was extracted from cell cultures using 1 mL of TriReagent (Molecular Research Center) per well cell culture, following the manufacturer's instructions. Quantification was carried out with a Nanodrop1000 (Thermo Scientific) and the quality of the RNA was checked with a Bioanalyzer (Agilent technologies). All RNA samples had a RIN value >7. Total RNA (2 μg) was used to synthesise cDNA with SuperScript III Transcriptase (Invitrogen) and oligo-dT primer (Promega).

### Measurement of PGE_2 _and PGD_2 _levels

Supernatants from stimulated cell cultures (triplicates) from 3 different fish were recovered, centrifuged and stored at -80°C until use. Measurement of PGE_2 _and PGD_2 _levels was completed with a monoclonal EIA according to the manufacturer's instructions. The prostaglandin kit detection limit was 8 pg/mL. Prior to prostaglandin determination supernatants were diluted five times in EIA assay buffer. The same macrophage cells were used to obtain total RNA for the determination of COX-2 and Prostaglandin D synthase gene expression as well as the supernatants for PGE_2_-PGD_2 _determination.

### Microarray analysis

The design of the microarray is described in detail elsewhere [40,41] and a full description of the platform and data presented in this manuscript are accessible through the public GEO depositories (accession number GPL6154 and GSE22330). The genes were selected by functional classes; random clones from common and subtracted cDNA libraries 1800 genes printed in six replicates each were compared with the known vertebrate proteins using BlastX; overall, the platform was enriched in a number of functional classes, such as immune response (236 genes), signal transduction (245 genes), receptor activity (126 genes), apoptosis (120 genes), cell cycle (70 genes), protein catabolism (90 genes), folding (70 genes), response to oxidative stress (39 genes), stress and defence response (145 and 105 genes, respectively), and chaperone activity (41 genes). Total RNA was extracted from cell cultures using 1 mL of TriReagent (Molecular Research Centre) per well, following the manufacturer's instructions, the quantity and integrity was analysed by Experion RNA StdSens Analysis Kit (Bio-Rad). Microarray analyses were conducted in pooled samples (see experimental design of microarray assay). A dye-swap design of hybridisation was applied. In analyses of infected immune cells, the non-infected cells were used as a control. Each sample was analysed with two slides. Scanning was performed with Alphascan (High Performance Dual-Laser Scanner for Microarray Slides from Alpha Innotech and images were processed with VisionLite (ThermoSpectronic). The measurements in spots were filtered by criteria I/B ≥ 3 and (I-B)/(SI + SB) ≥ 0.6, where I and B are the mean signal and background intensities and SI, SB are the standard deviations. After subtraction of mean background, locally weighted non-linear regression (Lowess) normalisation [42] was performed separately for each slide. To assess differential expression of genes, the normalised log intensity ratios were analysed with Student's t-test (p < 0.01). The Bayesian modification to the false discovery rate (FDR) was used to correct for multiple comparison tests, estimating the q-value for the set of differentially expressed genes [43]. The functional categories of Gene Ontology [44] were compared with regulated genes (*p *< 0.01) by the sums of ranks (Student t-test *p *< 0.05). The statistical significance of over-represented functional categories, showing the differential expression in the experiment grouped by functional classes compared with all genes an GO categories from the chip, was assessed using the Chi square test with Yates correction (*p *< 0.05).

### Real-Time quantitative PCR and validation

In order to verify microarray results, real-time PCR (qRT-PCR) was carried out. Two micrograms of the individuals RNA was used to synthesise cDNA with SuperScript III RNase Transcriptase (Invitrogen) and oligo-dT primer (Promega). As a house-keeping gene, 18S was amplified from the same cDNA samples. For different gene expression analysis specific primers were used (Additional file 1). Real-time PCR reactions were carried out in a 25 μL reaction with SYBR Green I (Stratagene) using a 1:25 dilution of the cDNA and 250 nM of primers. Quantitative qRT-PCR was performed using a Mx 3000P System (Stratagene) and quantification was done according to the Pfaffl method corrected for efficiency for each primer set [45]. Values for each sample were expressed as "fold differences", calculated relative to controls group and normalised for each gene against those obtained for the house keeping gene 18S.

### Experimental design

#### Microarray analysis

macrophage cell cultures isolated from 84 animals were stimulated with PGNs from *E.coli *O111:B4 and K12 strains and compared to parallel control cultures (without stimulation). Cell cultures were individually stimulated with both peptidoglycans for 1, 6 and 12 h (12 by PGN and time, n = 72), and 12 control cultures (total; n = 84). Individuals RNAs were grouped into three pools from 4 cell cultures for each time point (1, 6, and 12 h). The transcriptomic response was analysed by microarray assay, and divided in three experimental time points named early (1 h), median (6 h) and late stage (12 h). The analysis was carried out with common genes expressed within three replicate pools over the control (GDE one way ANOVA p > 0.01). The qRT-PCR validation assay was conducted with total RNA from late stage cell cultures.

#### Time Course

macrophage cell cultures isolated from 9 animals were stimulated with PGN O111:B4 and K12 during 0, 30 min, 1, 3, 6, and 12 h (10 μg/mL). The mRNA abundance of COX-2 (or prostaglandin endoperoxide synthase 2) and PTGDS was measured by qRT-PCR, prostaglandin release (PGE_2_-PGD_2_) were measured using a prostaglandin EIA assay (Cayman). Three individual replicates were made for each peptidoglycan stimulation. The control group was non-stimulated cell cultures (n = 3).

#### Dose-Response

macrophage cell cultures isolated from 9 animals were stimulated with PGN from the *E. coli *strains 0111:B4 and K12. The treatment was conducted overnight (12 h) with different concentrations, 0, 0.1 and 10 μg/mL, of PGNs. Expression of COX-2 and PTGDS mRNAs was measured by qRT-PCR, prostaglandin release (PGE_2_-PGD_2_) were measured using a prostaglandin EIA assay (Cayman). Three individual replicates were made for each peptidoglycan stimulation. The control group was non-stimulated cell cultures (n = 3).

### Statistical analysis

All statistical analysis was conducted with the software SPSS Statistic 17.0. The relationship between intensity of expression and time was examined and tested for significant differences between the PGNs with covariance analysis (ANCOVA) using the transcriptomic magnitude as co-variable, followed by one-way ANOVA analysis for up- or down regulated transcripts. The Student t-test was made to explore the difference between the expression registered in the microarray assay and the qRT-PCR (Additional file 2). Two-way ANOVAs were made to compare the differences between COX-2 and PTGDS expression and prostaglandin release in the time-course and dose-response assay.

## Results

### Global comparisons of the transcriptomic response to PGN (microarray analysis at 1, 6, and 12 hours)

**Figure 1:**
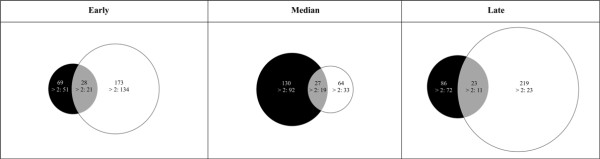
**Characterisation of the transcriptomic response**. A; Venn diagram representing mRNA transcripts differentially expressed over control during PGN-O111:B4 and PGN-K12 challenges throughout the time (early, median and late stage). The area of the circles is scaled to the number of transcripts (one way ANOVA *p *< 0.01) and the fold change (FC >2) expressed in each stage. Black circles: 69, 130, 86 number of transcripts differentially expressed under PGN-O111:B4 treatment. White circles: 173, 64, 219 number of transcripts differentially expressed under PGN-K12 challenge.

Microarray analyses were evaluated using a salmonid-specific targeted cDNA microarray containing 1800 cDNAs enriched with immune system related genes (SFA 2.0). Gene expression profiles obtained highlighted a marked contrast in the macrophage response to PGN purified from *E. coli *(PGN-O111:B4 and K12). Samples were taken over time early (1 h), median (6 h) and late stage (12 h) and separate one-way ANOVAs (*p *> 0.01) were conducted to identify differentially expressed transcripts over the control (GDE). Transcripts expressed within all three biological replicates were used to analyse changes for both treatment (PGN) and time stage (Additional files 3, 4, 5, 6, 7, 8). The kinetics of the response obtained from peptidoglycans derived from K12 or O111:B4 were significantly different in both transcript number (total number of differentially expressed transcript over the control, one-way ANOVA *p *< 0.01) and intensity (fold change FC >2) (Figure 1). In total 819 transcripts were differentially expressed (GDE) in both treatments over the control (all cDNAs expressed on the array), with 270, 221 and 328 in the early stage, median and late stages respectively (Figure 1, and Additional file 3, 4, 5, 6, 7, 8, 9). Stimulation with PGN-O111:B4 revealed a significant peak in intensity at the median stage (130 transcripts one-way ANOVA *p *< 0.01 and FC >2; 92) and a strong and intense response was maintained throughout (FC >2; 51, 92 and 72 at 1, 6 and 12 h respectively). In contrast PGN-K12 induced a significant diversity of transcripts (magnitude) over time, note a decrease at 6 h, where the response intensity although high at 1 h (FC >2; 134 transcripts) significantly decreased through time where late stage transcripts with FC >2 represent only 17% of the early stage total (Figure 1, Additional file 9). Regression analysis (up regulated genes ANCOVA, F_5, 68 _= 1.178 *p *> 0.05, followed by two-way ANOVA, F_2, 68 _= 27.124: *p *< 0.05; down regulated genes ANCOVA, F_5, 68 _= 2.303: *p *> 0.05, followed by two-way ANOVA, F_2, 68 _= 37.124: *p *< 0.05) (Additional file 10, and 11) highlights that a stronger induction of gene expression and likely more directed response is obtained with PGN-O111:B4 challenge.

### Qualitative comparisons of the transcriptomic response to PGN: Differentially expressed transcripts in early, median, and late stages of activation

#### Early stage

A higher number of induced transcripts were observed with PGN-K12 treatment in respect to PGN-B4 highlighting a common down-regulation of inflammatory processes (Table 1 and 2). Major differences could also be identified in ligand recognition where macrophages stimulated with PGN-K12 up-regulated BPI binding protein (BPI). In fish, BPI has been suggested to be involved in LPS binding and recognition [46] whereas PGN-B4 stimulation led to up-regulation of antigen-processing including MHC I, and MARCO. The alternative spliced form of MARCO, Cysteine-rich protein 1, that also recognises bacterial cell wall PAMP's was co-ordinately down-regulated [47]. Transcripts related to the inflammatory response were down regulated under both PGN challenges including for PGN-B4; N-acetylmuramoyl-L-alanine amidase (bactericidal activity), PGLYPR6 and peroxiredoxin (Table 1) and for PGN-K12; NF-κB inhibitor alpha-1 and arachidonate 5-lipoxygenase (Table 2). Microsomal glutathione S-transferase, a precursor for leukotriene and prostaglandin production [48] was down-regulated by both treatments. Interestingly, annexin A1-1 was strongly up-regulated (FC; 9.8) in response to PGN-K12. This transcript has been suggested to have anti-inflammatory activity due to its phospholipase A2 (essential for inflammatory prostaglandin production) inhibitory activity [49] (Table 2 and Additional file 6).

**Table 1:** Summary of selected transcripts expressed after challenges with PGN-O111:B4

	Early		Median		Late	
Antigen presenttion	Mean	SD	Mean	SD	Mean	SD
MHC class I heavy chain-1	4.92	2.35	3.27	1.67	n/s	n/s
Macrophage receptor MARCO	2.07	0.56	5.02	1.75	n/s	n/s
Cysteine-rich protein 1	-3.59	0.74	n/s	n/s	n/s	n/s
BPI binding protein	n/s	n/s	11.93	5.78	n/s	n/s
Cell adhesion and proliferation						
CD166	1.79	0.35	4.99	2.97	3.06	1.48
Cytokines and Chemokines						
C-C chemokine receptor type 3	4.25	3.47	n/s	n/s	n/s	n/s
Chemokine receptor CXCR4	n/s	n/s	-4.24	1.01	n/s	n/s
Cellullar defense response						
N-acetylmuramoyl-L-alanine amidase	-1.60	0.05	1.46	0.03	1.46	0.17
Peroxiredoxin 1-1	-2.98	1.42	1.30	0.76	1.74	0.30
Interleukin enhancer-binding factor 3	n/s	n/s	2.39	1.47	n/s	n/s
TNF decoy receptor	n/s	n/s	11.42	3.86	12.09	10.98
NF-kappaB inhibitor alpha-3	n/s	n/s	9.24	6.05	n/s	n/s
Myeloid differentiation primary response	n/s	n/s	n/s	n/s	1.56	0.29
Phosphotyrosine SH2 domain	n/s	n/s	n/s	n/s	2.86	1.17
Procathepsin L-1	n/s	n/s	4.11	1.67	n/s	n/s
Procathepsin L-2	n/s	n/s	3.47	1.28	n/s	n/s
Cathepsin B-2	n/s	n/s	3.36	2.29	n/s	n/s
Cathepsin D-2	n/s	n/s	3.99	0.25	n/s	n/s
Cathepsin C-1	n/s	n/s	3.28	1.16	n/s	n/s
Cathepsin C-2	n/s	n/s	5.14	5.30	n/s	n/s
MAPK/ERK						
Serine/threonine-protein kinase 2	n/s	n/s	5.03	2.65	n/s	n/s
MAPK/ERK kinase kinase 5-1	n/s	n/s	-1.68	0.33	n/s	n/s
C-Jun protein	n/s	n/s	n/s	n/s	3.99	1.62
MAPK/ERK kinase kinase 1-2	n/s	n/s	n/s	n/s	1.87	0.46
MAPK kinase 9-2	n/s	n/s	n/s	n/s	5.78	3.73

Transcripts represented were firstly selected for expression level (*p *< 0.01) and then implication in biological processes related to PGN stimulation (immune/inflammatory responses) during PGN-O111:B4. n/s: not signal, Mean: Fold expression average (n = 3), SD: standard deviation.

**Table 2:** Summary of selected transcripts expressed after challenges with PGN-K12.

	Early		Median		Late	
Antigen presenttion	Mean	SD	Mean	SD	Mean	SD
MHC class I heavy chain-1	1.6	0.3	3.8	1.1	4.1	3.9
BPI binding protein	3.4	2.5	n/s	n/s	1.5	0.6
Macrophage receptor MARCO	n/s	n/s	n/s	n/s	0.4	1.8
Cell adhesion and proliferation						
Fibronectin receptor beta	11.6	11.3	n/s	n/s	n/s	n/s
CD2 binding protein 1-1	2.9	1.1	n/s	n/s	n/s	n/s
Matrix metalloproteinase 9	2.0	0.7	n/s	n/s	-4.7	1.8
Cytokines and Chemokines						
Cytokine receptor gamma chain	1.7	0.5	n/s	n/s	n/s	n/s
CC chemokine SCYA110-1	n/s	n/s	n/s	n/s	1.2	0.1
Cellullar defense response						
TNF receptor associated factor 1	n/s	n/s	4.6	4.1	n/s	n/s
NF-kappaB inhibitor alpha-1	4.6	4.3	n/s	n/s	n/s	n/s
Cathepsin C-3	2.8	2.3	n/s	n/s	n/s	n/s
Cathepsin D-1	3.2	2.8	n/s	n/s	1.6	0.5
Cathepsin D-2	3.7	3.6	n/s	n/s	n/s	n/s
MAPK/ERK						
MAPK/ERK kinase kinase 6	1.7	0.3	2.1	0.5	n/s	n/s
Serine/threonine-protein kinase 2	2.9	2.2	n/s	n/s	-1.2	0.1
Tyrosine-protein kinase FRK	n/s	n/s	n/s	n/s	1.7	0.5
Tyrosine-protein kinase SYK	n/s	n/s	n/s	n/s	-2.0	1.4
Inflammatory response						
Annexin A1-1	9.8	9.4	n/s	n/s	1.4	0.3
Microsomal glutathione S-transferase 3	-1.6	0.2	-1.4	0.2	1.2	0.1
Arachidonate 5-lipoxygenase-1	-3.4	0.4	n/s	n/s	n/s	n/s
Prostaglandine D synthase	1.3	0.1	n/s	n/s	n/s	n/s
Angiotensin I converting enzyme	0.0	n/s	n/s	n/s	1.2	n/s
Cell homeostasis						
Metallothionein A	-4.4	1.1	n/s	n/s	1.4	0.2
Heat shock 27 kDa protein-1	2.8	0.8	n/s	n/s	-1.8	0.6
Heat shock 70 kDa protein 1	3.0	1.4	n/s	n/s	-1.7	0.4
Glutathione reductase	2.0	0.8	n/s	n/s	-2.2	1.0
Cellular metabolism						
Malate dehydrogenase, cytoplasmic	2.1	0.6	n/s	n/s	n/s	n/s
Glucose-6-phosphate isomerase-1	n/s	n/s	n/s	n/s	2.4	1.7
ATP synthase factor 6	2.4	1.7	n/s	n/s	n/s	n/s
Transcription						
Reverse transcriptase-like-2	2.4	1.7	-2.7	0.6	n/s	n/s
CCAAT/enhancer binding protein beta	n/s	n/s	7.0	2.6	1.4	0.5
Chromatin dis-assembly						
Transposase-15	-4.0	3.4	n/s	n/s	-1.3	0.3
Transposase-56	n/s	n/s	-3.4	0.5	n/s	n/s
G1/S-specific cyclin D2	-3.1	2.0	-3.6	1.4	1.5	0.4

Transcripts represented were selected for expression level (*p *< 0.01) and then implication in biological processes related to PGN stimulation (immune/inflammatory responses) during PGN-K12. n/s: no signal. Mean: Fold expression average (n = 3), SD: standard deviation.

#### Median stage

Of note at the median stage is that PGN-B4 induces a co-ordinated increase in pro-inflammatory and cellular defence activity with increased intensity (Table 1 and 2). Mediators of inflammatory prostaglandin production are up-regulated highlighted by increased arachidonate 5 lipoxygenase mRNA synthesis. In parallel, cathepsin transcripts (protease activity) (n = 6), PGLYPR6 (amidase) and the interleukin enhancer 3 mRNA (regulates interleukin production during infectious processes (e.g., [50]), were also up-regulated. PGN-K12 stimulation at this point is highlighted by a strong down-regulation of transcript diversity, including cell adhesion, defence response, cell homeostasis and metabolism, with almost all observed early stage transcripts returning to base-line conditions (Table 1 and 2). Potentially of importance is the up-regulation of the transcription factor CCAAT/enhancer binding protein β (C/EBP-β) mRNA by PGN-K12. C/EBP-β has been shown to be intimately linked to immune and inflammatory processes and regulates the transcription of the pro-inflammatory cytokine, interleukin-6. On the other hand, the tumour necrosis factor (TNF) decoy receptor, which inhibits apoptosis, and NF-κB inhibitor alpha-3 were strongly up-regulated in addition to an abrupt increase in BPI with PGN-B4.

#### Late stage

For PGN-B4 a defined response was observed after 12 h of stimulation where the prostaglandin endoperoxide synthase-2 (COX-2), and prostaglandin D synthase, both linked to the synthesis of inflammatory prostaglandins, were strongly up-regulated (Table 1). COX-2 (prostaglandin endoperoxide synthase-2) catalyses the conversion of arachidonic acid to prostaglandin (PGH_2_) [51,52], and prostaglandin D synthase (PTGDS) catalyses the conversion of PGH_2 _to prostaglandin D_2 _(PGD_2_) [53,54]. Signalling components for TLR pathways are also up-regulated by PGN-B4 including the MAPK pathways and myeloid differentiation primary response (MyD88) mRNA, an adapter protein between TLR and the transcription factor NF-κB. Interestingly these components plus the serine/threonine-protein kinase 2 are required to respond to microbial ligands [55]. TNF decoy receptor is maintained up-regulated highlighting the anti-apoptotic response of PGN-B4 activated macrophages. TNF-α is secreted into the culture medium as soon as 1 h after PGN treatment [MacKenzie et al, unpublished results]. In contrast to the strong inflammatory profile obtained for PGN-B4 the PGN-K12, response at 12 h appears related to biological themes associated with energy, protein metabolism and cellular homeostasis at a low level of intensity (Table 2 and Additional file 8). These results imply close similarities with those previously obtained for trout macrophages activated with crude LPS [56-59] suggesting a common recognition mechanism distinct to that observed for PGN-B4.

From transcripts identified as differentially expressed and significantly up- or down-regulated (one-way ANOVA *p *< 0.01) we selected sixteen transcripts from the late stage for qRT-PCR validation. All sixteen transcripts were significantly expressed between the two PGNs and significantly correlated when tested by qRT-PCR and Students-T test (*p *< 0.05); thereby confirming the microarray results. FC values obtain by microarray and qRT-PCR analyses are listed in the additional file 2 (Student T tests *p *> 0.05).

### Functional categories are associated with combinations of PGN and time parameters

**Figure 2:**
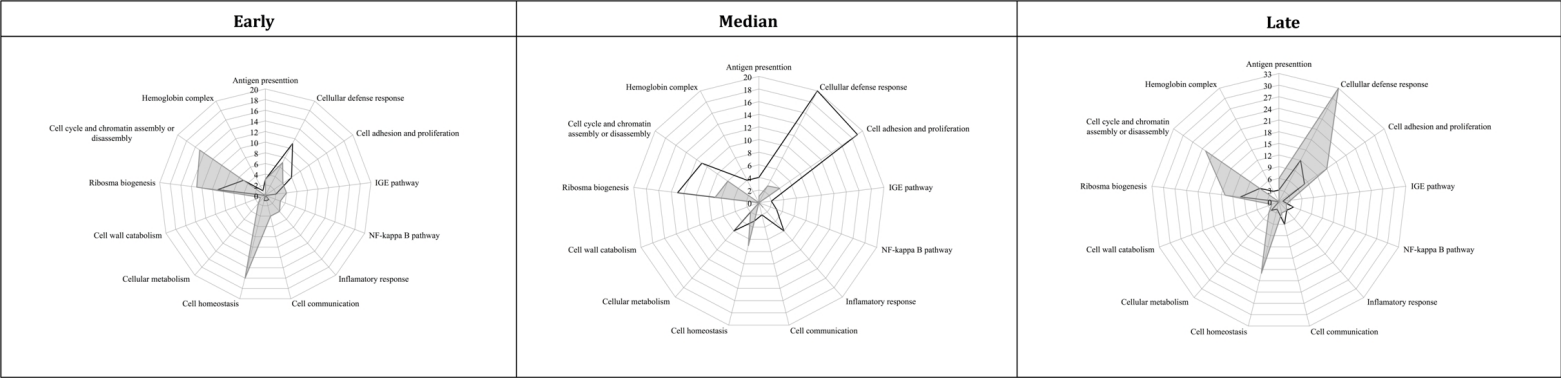
**Qualitative and quantitative representations of biological processes (GO) over represented during challenges**. Qualitative and quantitative representations of over expressed GO categories (Chi-square with Yates correction *p *< 0.05). The corners of the spider-web maps represent biological processes identified in the GO analysis. Different numbers of transcripts were grouped in each biological process. The continuous mark lines (black or grey) represent the different number of transcripts in each biological process. The differences in the shape of the GO pattern (continuous mark line) are due to divergence in the number of transcripts grouped to each Gene Class (biological process) under both PGN challenges; the black line shows the GO pattern for PGN-O111:B4 and the grey line shows the GO pattern for PGN-K12 treatment.

Analysis of function using GO annotations revealed that most over-expressed transcripts were related to the immune response and GO functional categories are specifically influenced by a combinatorial PGN-Time effect (Chi-square with Yates correction, *p *< 0.01, Figure 2). In the early stage, different GO categories expressed were PGN-dependent and include MHC class I receptor, lysozome, NF-κB cascade, peptidase activity, cell adhesion, ribosome, or chromatin assembly or disassembly (Figure 2). At the median stage the intensity of the PGN-B4 response is highlighted by a set of biological processes specifically associated to the immune response whereas only two GO classes, cell adhesion and negative regulation of cell proliferation, were represented with PGN-K12 (Figure 2). At the late stage an inverse correlation was observed where peptidase activity, complement activation, cell homeostasis, and mitochondrial electron transport were highly represented with PGN-K12 and NF-κB cascade, protein-MAPK cascade, and ribosome related to the PGN-O111:B4 response (Figure 2). Remarkably, cell wall catabolism was only observed with PGN-K12 and not during PGN-O111:B4 challenge (Figure 2).

### Characterisation of the prostaglandin response (time course and dose response of PGN challenges)

**Figure 3:**
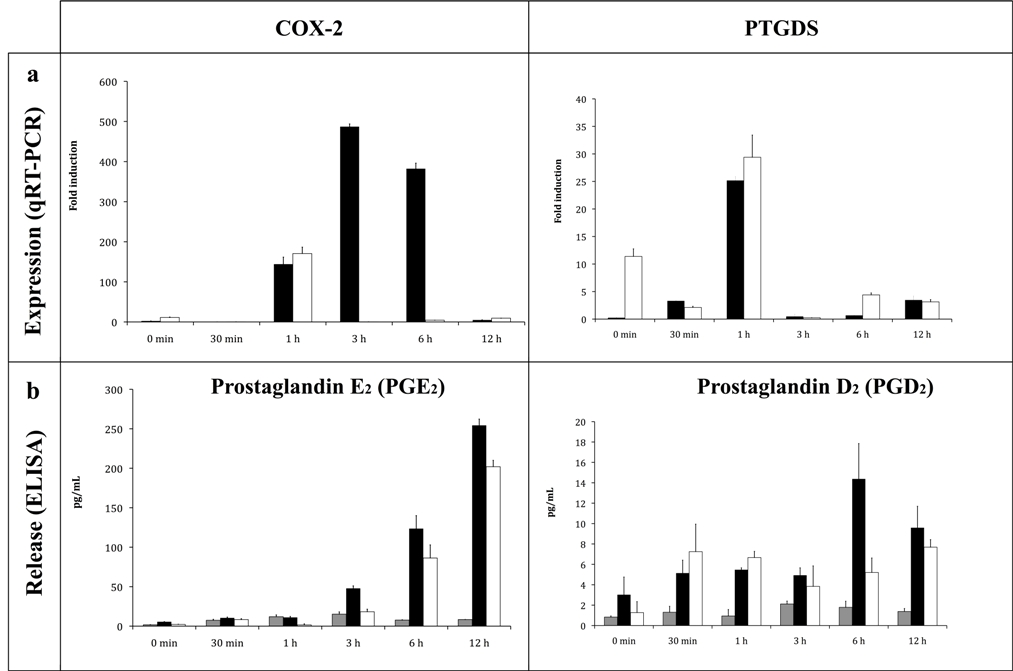
**Temporal characterisation of the prostaglandin response**. Time course response to macrophages stimulated during 0, 30 min, 1, 3, 6, and 12 h with 10 μg/mL of PGN O111:B4 and K12. Experiments were performed in independent groups of PGN-stimulated (n = 3) or control macrophage cultures (n = 9). a) COX-2 and PTGDS mRNA abundance over time in response to PGN-B4 (black bar) or PGN-K12 (white bar). Were observed significative differences in the mRNA abundance between the times and treatments (PGNs) in both genes (two way ANOVA p < 0.01). b) PGE_2 _and PGD_2 _release (pg/mL) stimulated by PGN-O111:B4 (black bars), PGN-K12 (white bars) and control (grey bars) into the culture medium (n = 3/treatment). Were observed significative differences in the release between the times and treatments (PGNs or control) by both prostaglandin (two way ANOVAs *p *< 0,01). The results are presents as fold change relative to 18S abundance and ± std deviation.

**Figure 4:**
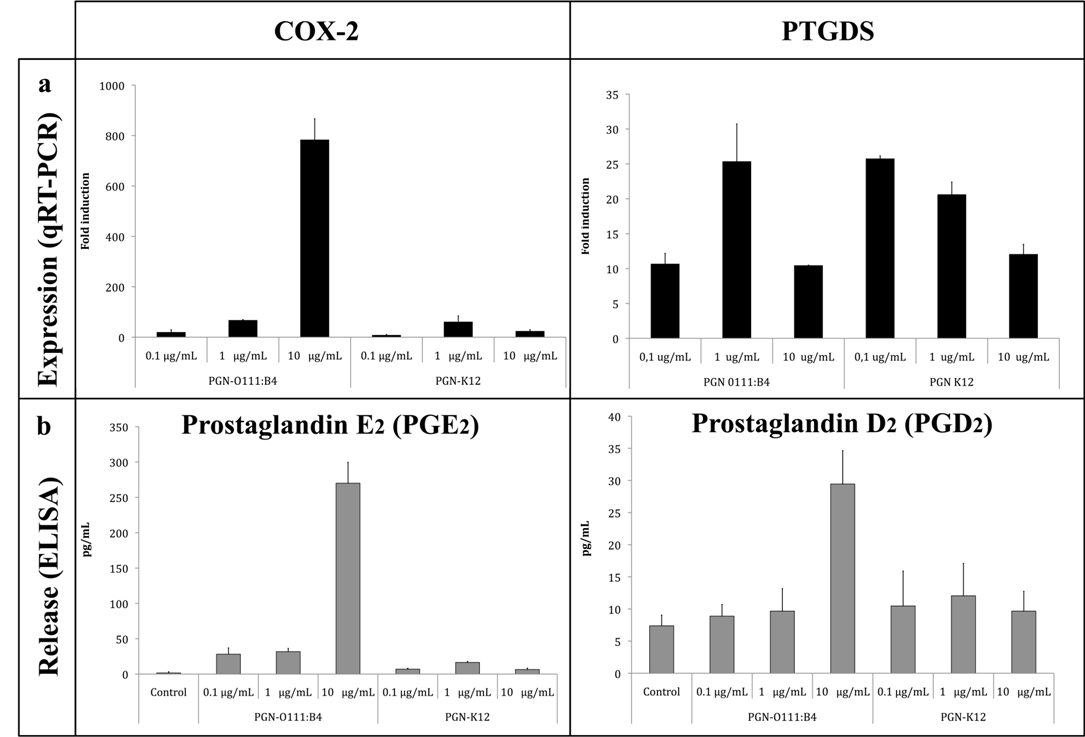
**Concentration dependence of the prostaglandin response**. Dose response (0.1, 1, and 10 μg/mL) of trout macrophages to PGN O111:B4 and K12 challenge. Experiments were performed overnight in independent macrophage cultures (n = 3). a) COX-2 and PTGDS mRNA abundance (black bar) in response to different doses of PGN-O111:B4 or PGN-K12 (0.1, 1, 10 μg/mL). Were observed significative differences in the mRNA abundance between different doses and treatments (PGNs) in both genes (two way ANOVAs *p *< 0.01). b) PGE_2 _and PGD_2 _release (pg/mL) into the culture medium (grey bars). Were observed significative differences in the release between doses and treatments (PGNs or control) by both prostaglandins (two way ANOVAs *p *< 0.01). The results are presented as fold change relative to 18S abundance and mean ± std deviation.

Both COX-2 and PTGDS were identified by microarray analyses as differentially expressed between the two PGNs, therefore we measured both PGE_2 _and PGD_2 _release into the culture supernatant and in parallel COX-2 and PTGDS mRNA abundance by qRT-PCR. Analyses were done both in respect to response to PGN-B4 and PGN-K12 over time (30 min, 1, 3, 6 and 12 h; Figure 3) and subsequently as a dose response (0.1, 1 and 10 μg/mL; Figure 4).

### Time course response assay (0, 30 min, 1, 3, 6, and 12 hrs of PGN challenges)

COX-2 mRNA expression is strongly regulated by PGN-B4 over time followed by a significant increase in PGE_2 _secretion into the culture medium. Stimulation with PGN-K12 results in an increase of mRNA abundance at 1 h (two way ANOVA, F_5, 35 _= 8.678, *p *< 0.05, Figure 3a, Additional file 12) and a more gradual accumulation of PGE_2 _in the culture medium in comparison with PGN-B4. The dynamics of PTGDS mRNA expression was time dependent (two way ANOVA, F_5, 35 _= 4.584, *p *< 0.05, Figure 3a, Additional file 12) showing changes a few minutes after stimulation with both PGNs (30 min) and a strong increase 1 h post-treatment (Figure 3a). The release of PGD_2 _was significantly different (increasing) in PGN-B4 treated macrophages 6 h after stimulation. Differences observed between PGE_2 _and PGD_2 _release are correlated to both time and treatment (two way ANOVA, F_10, 54 _= 4.553, *p *< 0.05, Figure 3b, Additional file 12) where PGD_2 _has a low response, concentrations in the range of 1-14 pg/mL, when compared with the PGE_2 _secretion, >200 pg/mL. PGE_2 _and PGD_2 _liberation patterns were strongly influenced by the interaction between PGN and time (two way ANOVA, F_10, 54 _= 2.522, *p *< 0.05, Figure 3b, Additional file 12).

### Dose response assay (0.1, 1, and 10 μg/mL of PGN O111:B4 and K12)

In dose response assays the expression pattern of COX-2 mRNA induction was both dose and PGN-dependent (two way ANOVA, F_5, 18 _= 5.824, *p *< 0.05, Figure 4a, Additional file 12). In figure 4a, a peak of COX-2 expression was registered at 10 μg/mL of PG-B4. Interestingly, PGN-K12 stimulation generated a lower expression of COX-2 mRNA (10 μg/mL; >50 fold) when compared to PGN-B4 although at a dose of 1 μg/mL fold changes are similar for both PGNs (Figure 4a). This is reflected in PGE_2 _liberation where 10 μg/mL of PGN-B4 generated a strong response (>600 fold increase; PGN-interaction, two way ANOVA, F_2, 48 _= 182.588, *p *< 0.05, Additional file 12) that correlated to increased COX-2 mRNA abundance and all other concentrations for both PGNs induced similar responses (>50 fold). The liberation pattern of PGD_2 _was significantly dependent upon PGN type, and showed a single increase at 10 μg/mL with PGN-B4 (Two way ANOVA, F_2, 48 _= 4.588, *p *< 0.05, Additional file 12). Surprisingly this is not mirrored in PTGDS mRNA abundance levels where PTGDS mRNA is significantly up-regulated by PGN-K12 at 0.1 and 1 μg/mL and PGN-B4 at 1 μg/mL (Figure 4b, Additional file 12).

## Discussion

In recent studies on trout macrophages, peptidoglycan (PGN-B4) was identified as a major pro-inflammatory component of crude LPS preparations in which TLR4 and canonical TLR2 signalling pathways were discarded as potential recognition systems for peptidoglycans [9]. As structural differences in PGN peptide moieties from different bacterial-strains have been shown to modulate host responses in both Drosophila and mammals [32,36,38] we investigated, a priori with targeted microarray analysis, the effects of two different PGNs from different strains of *E. coli*, O111:B4 and K12. These different serological features have been shown to affect the host immune response [60]. A systematic dissection of the impact of (combinations of) culture parameters (time and treatment) revealed a significant re-modelling of the trout macrophage transcriptome highlighting the divergence of the response to the two different PGNs (PGN-B4 vs. PGN-K12). As there were no other known variables, the differences in the transcriptomic profile are assumed to be solely due to the structure of the different PGNs and therefore differential recognition of those by the macrophages. This assumption is supported by the variation in transcript number (Figure 1a, 2), their intensities (Figure 1a,b), and diversity (Table 1 and 2). In fish, modifications in the transcriptomic profile have been observed in response to environmental changes, stress and maintenance of the steady state of transcriptional activity [61,62], or bidirectional transcriptomic remodelling to inflammatory stimuli [56,63-67]; however, our data emphasises that macrophages respond differentially to highly similar bacterial PGNs resulting in a directed response i.e. prostaglandin release or a more generalised 'state of activation'.

In fish, the shift from a steady state to a functional inflammatory state, i.e. secretion of pro-inflammatory cytokines or PGE_2, _in trout macrophages stimulated with crude LPS preparations has been shown to be driven mainly by gram negative PGN, where DNA and RNA and ultra-pure LPS preparations are unable to induce mRNA expression of pro-inflammatory cytokines [9,68]. Our microarray analysis identified differential regulation of both prostaglandin D-synthase (PTGDS) and prostaglandin endoperoxide synthase-2 (COX-2) that are directly involved in eicosanoid production; PGD_2 _and PGE_2 _respectively [51,53] (Table 1 and 2). COX-2 is regulated in macrophage/monocyte cell types and is responsible for inflammatory prostaglandin, PGE_2_, synthesis from arachidonic acid, and is involved in cellular or tissue damage generated in acute and/or chronic inflammatory states [69]. PTGDS metabolises PGH_2 _to PGD_2_, [53,54,70,71] where PGD_2 _plays a role during the injury process as vasodilator/constrictor or as potent inflammatory mediator [72,73]. However, the action of PGD_2 _in fish as a mediator of the immune response is undefined. Downstream analyses, qRT-PCR and prostaglandin release, of both COX-2 and PTGDS mRNA regulation and PGH_2 _and PGD_2 _concentration in supernatants reveals a strong correlation, both time and dose-dependent, between PGN-type (B4 vs K12), mRNA abundance and inflammatory outcome (Figure 4). PGN-B4 is clearly a more potent regulator of the COX-2 mRNA/PGE_2 _pathway where the activation threshold for *de novo *synthesis of COX-2 is 10 μg/mL of PGN-B4. Interestingly this threshold concentration has also been observed on numerous occasions for pro-inflammatory cytokine mRNA synthesis in trout macrophages [9,39,46,68]. On the other hand, PTGDS mRNA synthesis appears as more dose sensitive for both PGNs with a similar temporal expression pattern suggestive of a different signal transduction mechanism. However PGN-B4 stimulation at 10 μg/mL results in higher PGD_2 _secretion. The regulation and biological effects of PGE_2 _and PGD_2 _secretion in inflammatory responses in fish clearly warrant more investigation.

In Drosophila the biological activity of a large panel of natural and synthetic DAP-PGN showed significant variability in their stimulatory capacity and immune response [74] and PGRP (peptidoglycan recognition protein) deficient *Drosophila *are more susceptible to bacterial infections [75]. In human monocytes exposed to synthetic muropeptides (peptide moiety of PGNs), TNF-α mRNA expression and release was highly dependent upon structural modifications between peptides [38]. Thus inflammatory outcomes are modified in accordance to sensitivity to peptidoglycan structure. Such sensitivity is likely conferred by the participation of different PRRs, PAMP-PRR interactions or the accumulative signalling intensity (i.e. threshold) of the group of PRRs involved in recognition.

Peptidoglycan recognition in mammals is mainly facilitated by three different PRR families; TLR2 (gram positive peptidoglycan), NOD2 and PGRPs all of which can bind peptidoglycans [35,75,76]. TLR2 has been described in fish species [77] although stimulation with lipoprotein (Pam3CSK4), a classical TLR2-ligand, does not stimulate an inflammatory response in our macrophage model [9]. However MyD88, an adaptor molecule involved in the classical Drosophila or mammalian Toll signalling cascades, which together with the receptor associated kinase (IRAK) and TNF activated factor (TRAF6) allow NF-κB translocation to the nucleus (promoting expression of inducible inflammatory cytokines such as TNF-α) during gram-negative bacterial infection [46,78-80] was specifically up-regulated during PGN-B4 stimulation. This suggests TLR involvement in the PGN-mediated inflammatory response in trout macrophages. Concerning PGRPs, PGRP-2, -5 and -6, have been shown in the zebrafish to play an essential role in defence during bacterial infections [30] and in the trout PGRP-2 responds to PGN-B4 [9]. In this study we also identified PGLYRP-6 (up-regulated; PGN-B4) suggesting that the PGRPs also play a role in specific-PGN recognition and this may be conserved throughout the fishes.

In contrast to the specific directed response obtained from PGN-B4 stimulation, PGN-K12 did not elicit a clear functional response at the level of the macrophage transcriptome or release of inflammatory mediators. A wide diversity of transcripts were activated although at a relatively low level. These results are similar to those previously observed for stimulation with crude LPS preparations in trout macrophages [[56], Boltaña et al., unpublished data] where both preparations can stimulate the release of TNF-α into the culture medium [[68], Roher et al., unpublished data]. Interestingly, TNF receptor associated factor 1 was specifically induced by PGN-K12. This transcript encodes a receptor-protein involved in the activity of apoptotic pathways mediated by TNF-α [80-82], however, we did not detect apoptosis during the experimental period [MacKenzie et al, unpublished data]. Moreover, the gene ontology category cell wall catabolism was consistently over-expressed throughout PGN-K12 treatment (Figure 2a,b) supporting the existence of a strong transduction signal generated by PGN-K12.

## Conclusions

Our data highlights the significant differences observed in macrophages responding to two PGNs derived from different serotypes of the same bacteria. Responses at the level of the transcriptome and the inflammatory outcome (prostaglandin synthesis) highlight the different sensitivity of the macrophage to slight differences (serotype) in peptidoglycan structure. Such divergent responses are likely to involve differential receptor sensitivity to ligands or indeed different receptor types. Such changes in biological response will likely reflect upon pathogenicity of certain serotypes and the development of disease.

## Abbreviations

PAMPs: pathogen-associated molecular patterns; PGN: peptidoglycans; PGRPs: peptidoglycan recognition proteins; GDE: differentially expressed genes; COX-2: Cyclooxygenase 2; PTGDS: prostaglandin D synthase.

## Authors' contributions

SM, SB and FG participated in the conception of the experimental design. SB and SM carried out the analysis. SB, FRL and DM performed microarray experiments. SB edited expression data, carried all statistical analysis and validated array data with qRT-PCR. SB and SM wrote the manuscript and FG corrected it. All listed authors edited the manuscript. All authors read and approved the manuscript.

## Supplementary Material

Additional file 1 **Specific primers used for quantitative qRT-PCR (sequence and accession number)**. Click here for file

Additional file 2 **Comparison of expression data for selected transcripts obtained from microarray analyses and qRT-PCR validation**. The results are presented as fold change relative to 18S abundance and mean ± std deviation. Click here for file

Additional file 3 Description of PGN (O111;B4) regulated transcripts/genes over the control (all cDNAs on the array) at early stage (1 h) Click here for file

Additional file 4 **Description of PGN (O111;B4) regulated transcripts/genes over the control (all cDNAs on the array) at median stage (6 h)**. xls Click here for file

Additional file 5 **Description of PGN (O111;B4) regulated transcripts/genes over the control (all cDNAs on the array) at late stage (12 h)**. xls Click here for file

Additional file 6 **Description of PGN (K12) regulated transcripts/genes over the control (all cDNAs on the array) at early stage (1 h)**. Click here for file

Additional file 7 **Description of PGN (K12) regulated transcripts/genes over the control (all cDNAs on the array) at median stage (6 h)**. Click here for file

Additional file 8 **Description of PGN (K12) regulated transcripts/genes over the control (all cDNAs on the array) at late stage (12 h)**. Click here for file

Additional file 9 **Quantitative summary of transcripts/genes differentially expressed over the control in both treatment and stages**. Click here for file

Additional file 10 **Relationship between intensity and magnitude of transcriptomic response in up (a) and down (b) regulated genes at different time stages during the PGNs challenge**. The horizontal abscises (magnitude) show the number of transcripts grouped in biological processes expressed in both treatments as: Antigen presentation, Cell adhesion and proliferation, Cytokines and Chemokines, Cellullar defense response, MAPK/ERK, Inflammatory response, Cell homeostasis, Transcription. The vertical abscises (intensity) show fold change mean (FC: intensity) of the transcripts grouped in each biological process. The black circle and the blue slope represented the fit generated by the intensity and magnitude of the transcriptomic response under PGN-O111:B4 treatment. The white circle and the red slope represented the fit generated by the intensity and magnitude of the transcriptomic response under PGN-K12 treatment. Transcriptomic profiles were highly ranked dependent upon PGN-type (two-way ANCOVA on transcriptomic magnitudes of respective intensities α = 0.05; n = 68). Click here for file

Additional file 11 Summary of ANCOVA analysis for common slope of regression and adjusted means examining differences in intensity and magnitude of the transcriptomic response at different times (1, 6 and 12 h; n = 68) Click here for file

Additional file 12 **Summary of multiple comparisons of two way ANOVAS**. Click here for file
