# Supplementary figures and images for: Divergent responses to peptidoglycans derived from different E. coli serotypes influence inflammatory outcome in trout, Oncorhynchus mykiss, macrophages
Source: BMC Genomics. 2011 Jan 14;12:34. doi: 10.1186/1471-2164-12-34 (PMC3087353; doi:10.1186/1471-2164-12-34)

Early

Median

Late

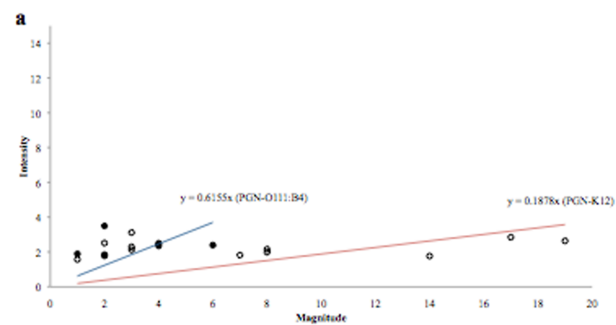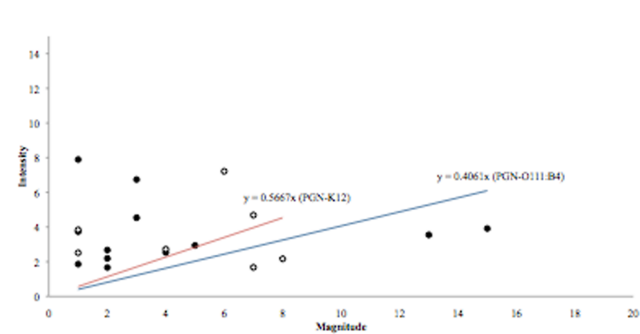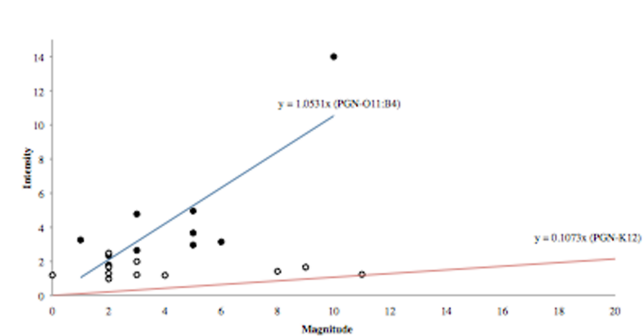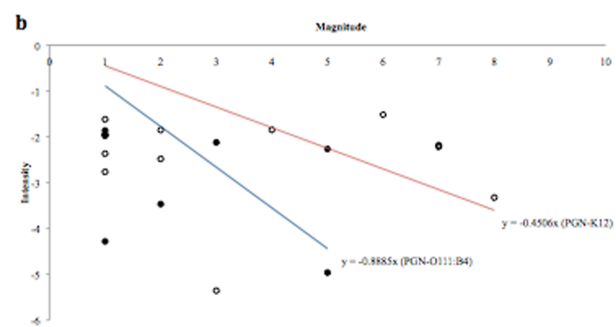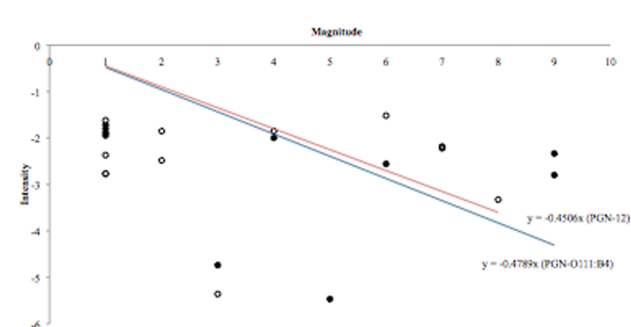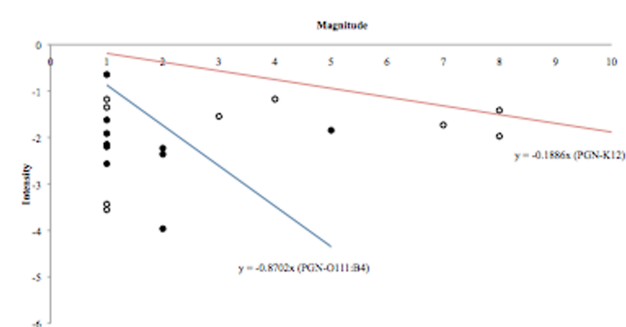

Supplement: Additional file 10 — Relationship between intensity and magnitude of transcriptomic response in up (a) and down (b) regulated genes at different time stages during the PGNs challenge. The horizontal abscises (magnitude) show the number of transcripts grouped in biological processes expressed in both treatments as: Antigen presentation, Cell adhesion and proliferation, Cytokines and Chemokines, Cellullar defense response, MAPK/ERK, Inflammatory response, Cell homeostasis, Transcription. The vertical abscises (intensity) show fold change mean (FC: intensity) of the transcripts grouped in each biological process. The black circle and the blue slope represented the fit generated by the intensity and magnitude of the transcriptomic response under PGN-O111:B4 treatment. The white circle and the red slope represented the fit generated by the intensity and magnitude of the transcriptomic response under PGN-K12 treatment. Transcriptomic profiles were highly ranked dependent upon PGN-type (two-way ANCOVA on transcriptomic magnitudes of respective intensities α = 0.05; n = 68). [file 1471-2164-12-34-S10.PDF]
